# Modifying the course of asthma: mechanisms and strategies for clinical remission

**DOI:** 10.1097/ACI.0000000000001129

**Published:** 2025-12-01

**Authors:** Benedetta Bondi, Diego Bagnasco, Fulvio Braido

**Affiliations:** a Respiratory and Allergy Clinic, IRCCS Ospedale Policlinico San Martino; b Department of Internal Medicine (DIMI), University of Genoa, Genoa, Italy

**Keywords:** asthma, biologics, clinical remission, precision medicine, severe asthma

## Abstract

**Purpose of review:**

Asthma management is ongoing a paradigm shift from symptom control and exacerbation prevention toward the more comprehensive goal of clinical remission. This review is timely because biologic therapies, precision medicine, and improved understanding of immunopathological mechanisms have made remission a realistic therapeutic goal. By integrating clinical, functional, and biological outcomes, remission offers a more comprehensive framework for assessing long-term disease control.

**Recent findings:**

Recent evidence demonstrate that biologic drugs, such as Mepolizumab, Omalizumab, Dupilumab, Benralizumab, and Tezepelumab, allow clinical remission to be achieved in many patients affected by severe asthma particularly those who show a phenotyping polarized toward T2-High. Lifestyle change, particularly weight loss and smoking cessation, early intervention, and the use of allergen immunotherapy may increase the chances of achieving remission. Real-world data confirm that remission rates vary depending on the definition applied, going from clinical to complete remission, highlighting the lack of a universally shared definition of remission and the need for standardized criteria.

**Summary:**

Clinical remission in asthma is now a feasible target. Achieving this goal requires a multidimensional approach that integrates biologics, early treatment, comorbidity management, and lifestyle interventions. Standardized definitions and biomarkers are essential to guide therapeutic decisions and predict long-term outcomes.

## INTRODUCTION

Asthma is a heterogeneous, chronic airways inflammatory disease that affects over 300 million individuals worldwide and contributes substantially to morbidity, healthcare costs, and reduced quality of life [1]. Historically, the focus of asthma treatment were symptom control, prevention of exacerbations, and maintenance of lung function [2]. In parallel with the shift from the “one size fits all” approach to precision and personalized medicine -- based on a thorough understanding of the pathophysiological mechanisms driving the disease -- the concept of clinical disease remission has emerged as a more holistic therapeutic target in asthma management.

In the past, the therapeutic management of asthma was predominantly centered on symptomatic relief, primarily targeting bronchoconstriction and acute manifestations using short-acting bronchodilators and oral corticosteroids (OCS). The advent of inhaled corticosteroids subsequently marked a paradigm shift, redirecting the therapeutic approach toward achieving sustained disease control and minimizing the frequency of exacerbations [1]. More recently, growing attention has been devoted to strategies that not only ensure symptom control but also optimize long-term outcomes and mitigate future risk [3].

However, this disease-modifying potential remained largely elusive until the recent advent of precision medicine and biologic therapies. The introduction of biologics has made it possible to achieve effective disease control even in the most challenging cases of patients affected by severe asthma, presenting as disease-modifying drugs. Nevertheless, clinical trials have traditionally concentrated on individual outcomes in isolation, such as reduction of exacerbations, sparing of OCS, improvement in quality of life, lung function, or symptoms. With the emergence of the concept of Clinical Remission, however, attention has shifted toward a more integrated perspective encompassing all these dimensions. Increasingly, the goal has shifted toward not just controlling asthma but modifying its trajectory to achieve sustained clinical remission. 

**Box 1:**
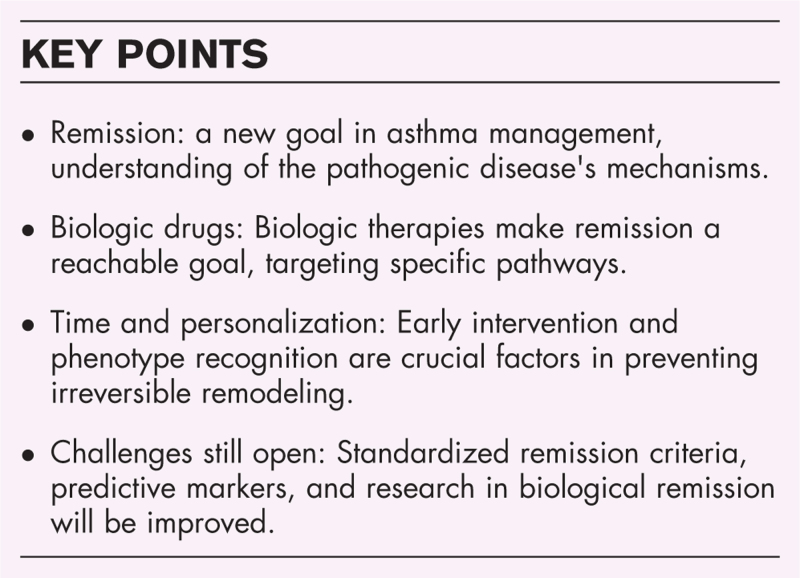
no caption available

**FIGURE 1:**
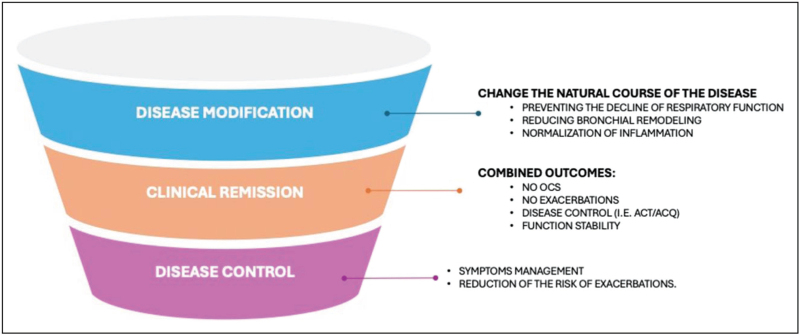
Differences between disease modification, clinical remission, and disease control.

Clinical remission must be differentiated booth from disease control and disease modification. Traditionally, clinicians aimed to achieve disease control, focusing on the reduction or absence of exacerbations and residual symptoms, assessed using tools such as Asthma Control Questionnaire (ACQ) or Asthma Control Test (ACT). Asthma control is usually assessed in two domains: current symptom control (e.g., frequency of daytime symptoms, night waking, reliever use, activity limitation) and future risk of adverse outcomes (e.g., exacerbations, lung function decline, medication side effects). Clinical remission, on the other hand, represents a more ambitious clinical goal, reaching the simultaneous presence of prolonged absence of symptoms, no exacerbations, no use of OCS, and stable lung function. Another crucial difference is the timing of observation, usually lower in control, and more prolonged in remission (at least 12 months). Finally, disease modification involves the presence of a therapy capable of modifying the natural history of asthma, preventing functional decline and disease progression, not only by acting on symptoms, but also by acting on the underlying pathogenic mechanisms (Fig. 1).

The evolving conceptual framework of asthma management thus includes three interrelated objectives: inflammatory suppression, functional preservation, and structural modulation of the airways. These are made possible by mechanistic insights into asthma's immunopathology – especially type 2 (T2) inflammation – and the development of targeted therapies. This review synthesizes current knowledge on the mechanisms that facilitate asthma remission and highlights strategic clinical interventions capable of modifying the disease course.

## PATHOPHYSIOLOGICAL BASIS FOR REMISSION IN ASTHMA

Asthma is not a monolithic disease but a collection of endotypes with distinct underlying immunological mechanisms. T2-high asthma is driven predominantly by eosinophilic inflammation mediated through IL-4, IL-5, and IL-13, while T2-low asthma may involve neutrophilic or paucigranulocytic inflammation, often resistant to corticosteroids.

The hallmarks of asthma pathophysiology include airway inflammation, bronchial hyperresponsiveness, mucus hypersecretion, and remodeling of airway structures such as epithelial cells and smooth muscle. These mechanisms are dynamic and potentially reversible in early stages, but chronic inflammation can lead to fixed airflow limitation. By intervening early and effectively modulating key inflammatory pathways, particularly in T2-high phenotypes, clinical remission becomes a feasible outcome.

The key immunologic targets include:

(1) IL-5 and IL-5Rα: Central in eosinophil maturation and survival.
(2) IL-4 and IL-13: Involved in B-cell class switching to IgE and goblet cell metaplasia.
(3) IgE: Key mediator in allergen-induced responses.
(4) TSLP and IL-33: Alarmins that initiate T2 inflammation at the epithelial level.

Histopathological studies demonstrate that anti-IL-5 and anti-IL-4R therapies significantly reduce eosinophilic infiltration and mucus gland hyperplasia. Suppression of these pathways not only mitigates symptoms but may reverse or halt structural remodeling, a critical aspect of modifying the disease course.

## CLINICAL STRATEGIES TO ACHIEVE REMISSION

### Biologic therapies

Biologic agents have revolutionized the treatment landscape for severe asthma, particularly in T2-high endotypes. Omalizumab was aimed primarily at forms of allergic asthma; it is an anti-IgE monoclonal antibody, able to bind the above-mentioned circulating immunoglobulins by inhibiting interactions with other cells (i.e., mast cells, eosinophils, and basophils). Subsequently, attention turned to interleukin-5 and its role in the development, growing and maturation of eosinophils, as target option for eosinophilic asthma. For these reasons, anti-IL-5 drugs such as Mepolizumab and Reslizumab were developed. Another time against eosinophils, but this time with an action on their IL-5 receptor (IL-5r) Benralizumab, was developed, tested, and marketed [4]. Further drug, Dupilumab is an anti-IL-4-R antibody that inhibits this cytokine receptor, interfering both with the signal transduction of IL-4 and IL-13. Among its various effects, dupilumab has the role of modulating the inflammatory response associated with B cells, IgE, and consequently, the cascade of events triggered by allergens [5,6]. Finally, Tezepelumab, a fully human IgG2λ mAb, binds TSLP, preventing its interaction with the TSLP receptor complex and thereby inhibiting multiple downstream inflammatory pathways. It acts as an upstream regulator, activating dendritic cells and group 2 innate lymphoid cells, which in turn drive type 2 (T2) inflammation and airway remodeling. Numerous data from clinical trials and real-world evidence indicate that Tezepelumab is effective also in non-T2 inflammation, although the best response to this drug is observed in T2 patients (Table 1).

**Table 1:** Biologic agents and clinical remission data

Biologic	Target	Population	Remission rate (%)	References
Benralizumab	IL-5Rα	Severe eosinophilic,+ CRSwNP	47.5%	Wechsler *et al.;* Pini *et al.* [7,8^▪^]
Mepolizumab	IL-5	Eosinophilic asthma	28–35%	Thomas *et al.;* Bagnasco *et al.* [9,10^▪^]
Dupilumab	IL-4Rα	Eosinophilic + allergic + CRSwNP	30–40%	Bagnasco *et al.;* Caminati *et al.* [11^▪^,^12^]
Omalizumab	IgE	Allergic asthma	20–25%	Thomas *et al.;* Casale *et al.;* Tiotiu *et al.* [9,13,14]
Tezepelumab	TSLP	Broad, T2-high	55%	Gates *et al.* [15]

Benralizumab's near-complete eosinophil depletion through antibody-dependent cytotoxicity provides rapid and sustained inflammation control. In the ANDHI and PONENTE trials, high rates of remission were observed, especially in those with comorbid CRSwNP [7,8^▪^]. Dupilumab, with its dual blockade of IL-4 and IL-13 signaling, has shown broader efficacy in T2-high patients, including those with elevated FeNO and IgE. Recent real-world data from Italy and the Netherlands demonstrate remission rates nearing 40%, particularly in patients with nasal polyposis and elevated eosinophils [11^▪^,^12^]. Mepolizumab continues to show a solid remission profile in eosinophilic patients, with consistent biomarker reductions and reduced exacerbation frequency [9,10^▪^].

Furthermore, a recent systematic review and meta-analysis evaluating remission across all biologics highlighted that CRSwNP is among the most powerful predictors of achieving clinical remission, with patients exhibiting higher ACT scores and greater biomarker normalization [16^▪^,^17^]. This reinforces the idea that comorbidities not only shape the inflammatory milieu but also enhance therapeutic outcomes.

### Early intervention and disease modification

Timing is a crucial factor in modifying disease course. Delayed initiation of biologics may allow irreversible remodeling and airflow limitation. Retrospective cohort studies from the UK Severe Asthma Registry showed that patients with a disease duration under 5 years were twice as likely to achieve remission upon biologic initiation compared to those with longer-standing disease [18].

Prospective trials now explore the concept of early intervention. The SYGMA trials with budesonide-formoterol demonstrated that even mild asthma benefits from anti-inflammatory treatment, suggesting a window of opportunity to modulate disease progression.

### Allergen immunotherapy

Allergen immunotherapy (AIT) can induce long-term immune tolerance by skewing immune responses from Th2 to Th1 and increasing regulatory T-cell populations. Long-term studies have reported sustained asthma remission in 10–15% of pediatric patients post-AIT discontinuation [19,20]. However, its role in adult and severe asthma is limited by safety concerns and variable efficacy.

### Environmental and lifestyle interventions

Environmental triggers, obesity, and smoking influence asthma control and remission. In obese patients, structured weight loss programs (>10% body weight) lead to significant improvements in lung function and symptom control [21].

Smoking cessation reverses corticosteroid resistance, and air pollution mitigation improves FeNO and eosinophil profiles [22]. A multifactorial lifestyle program implemented in a Dutch cohort achieved clinical remission in 38% of obese asthmatic individuals over 12 months [22].

## THE ROLE OF COMORBIDITIES IN REMISSION POTENTIAL

Comorbid conditions not only exacerbate asthma but also alter its immunological phenotype and treatment response. CRSwNP is a prototypical T2-high comorbidity strongly predictive of biologic response. In multiple biologic registries, CRSwNP presence doubled the odds of remission [7,23].

Importantly, recent evidence [16^▪^,^24^,^25^] has elucidated the role of CRSwNP in enhancing response to biologic treatment. Patients with both asthma and CRSwNP treated with Dupilumab or Benralizumab showed more consistent biomarker control, improved ACT scores, and higher sustained remission rates, compared to those without this comorbidity. CRSwNP may thus act as a phenotypic enhancer of T2 inflammation, thereby amplifying the efficacy of T2-targeting agents.

Obesity, OSAS, and GERD are associated with T2-low inflammation and poorer outcomes. Obese patients exhibit altered adipokine profiles, systemic inflammation, and decreased biologic efficacy. GERD contributes to chronic cough and upper airway inflammation. OSAS amplifies nocturnal hypoxia and airway inflammation.

Integrated management strategies – including CPAP for OSAS, proton-pump inhibitors for GERD, and bariatric interventions – can improve asthma outcomes and enhance remission likelihood.

## DEFINING AND MEASURING ASTHMA REMISSION

Standardized definitions of remission are essential. A widely used composite includes:

(1) No exacerbations in 12 months;
(2) No OCS use;
(3) ACQ 1.5 or less or ACT at least 20;
(4) FEV1 at least 80% predicted.

Some studies add biomarker control (eosinophils <150/μl, FeNO <25 ppb) to define biological remission. Imaging studies using HRCT have shown reduced airway wall thickness and mucus plugging in patients achieving sustained control with biologics [26^▪^,^27^].

Emerging digital tools, including wearables and home spirometry, offer real-time monitoring of disease control and early identification of relapse.

### Real-world evidence and definitions of remission

Real-world studies provide valuable insights into the effectiveness of biologics and other strategies in diverse populations. These studies often include patients with comorbidities, polypharmacy, and varying adherence, factors not typically represented in clinical trials. Registries such as the International Severe Asthma Registry (ISAR) and real-world cohort studies from Europe and the U.S. have demonstrated that remission rates may vary significantly depending on the definition used and patient characteristics.

**FIGURE 2:**
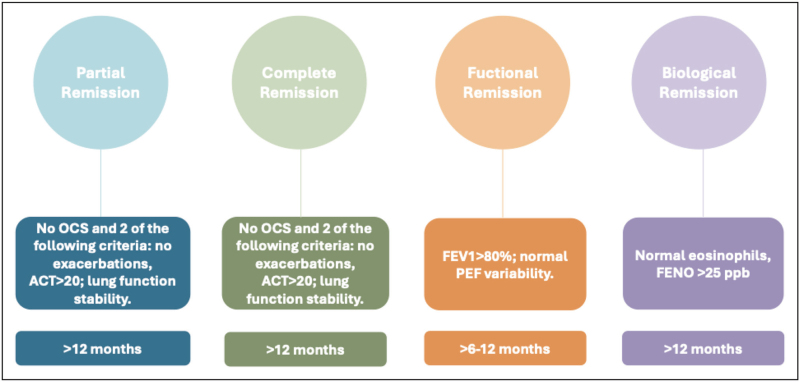
Models and criteria for asthma remission.

Multiple definitions of asthma remission exist, ranging from clinical to immunological remission (Table 2). Clinical remission usually refers to a state with no symptoms, no exacerbations, and no need for rescue medication for at least 12 months. Functional remission includes normalization or significant improvement in lung function, while complete remission also considers biomarker normalization and absence of airway inflammation (Fig. 2).

**Table 2:** Models and criteria for asthma remission

Type of remission	Criteria	Typical assessment time	References
Partial clinical remission	No OCS, and 2 of the following criteria: no exacerbations; ACT>20; Lung function stability	≥12 months	*Canonica et al.* [28];
Clinical remission	No OCS, and all the following criteria: no exacerbations; ACT>20; Lung function stability	≥12 months	Canonica *et al.* [28];
Functional remission	FEV1 >80%, normal PEF variability	≥6–12 months	Canonica *et al.;* Menzies-Gow *et al.;* Lommatzsch *et al.* [16^▪^,^29^,^30^]
Biological remission	Normal eosinophils, FeNO <25 ppb	≥12 months	Canonica *et al.;* Thomas *et al.* [16^▪^,^31^];

These varying definitions underscore the importance of a standardized framework to assess remission and tailor therapy accordingly. Among the factors limiting the applicability of this concept in clinical practice is undoubtedly the heterogeneity of definitions of remission, not only in terms of the necessary criteria but also in terms of the observation period (some authors suggest that a period of at least 12 months is necessary, while others limit it to 6 months). This heterogeneity is also present in patients, as they have a variety of clinical characteristics, both in relation to the asthma itself (T2 high vs. T2 low) and in terms of behavioral habits, biomarkers, and comorbidities. These latter elements, in fact, are decisive in achieving remission [18,32,33].

## REAL-WORLD OUTCOMES AND REMISSION RATES

Real-world evidence (Table 3) confirms the potential for remission:

(1) Benralizumab: remission in 69.5% at 12 months, 77.50% at 24 months, and 68.63% at 36 months [8^▪^].
(2) Omalizumab (PROSPERO): remission in 21.8% at 36 months [13];
(3) Tezepelumab (multicenter; NAVIGATOR and DESTINATION): 55% remission in patients with T2-high asthma [15];
(4) Dupilumab (Italy): remission in 54%, particularly the ones with CRSwNP [11^▪^];
(5) Mepolizumab (Italy): remission in 52% at 12 months, 41% at 24 months, and 73% at 36 months [10^▪^].

**Table 3:** Real-world remission outcomes across biologics

Biologic	Population	Duration	Remission Rate (%)	References
Benralizumab	Severe, OCS-dependent	12–24–36 months	43.4%	Pini *et al.* [8^▪^]
Omalizumab	Allergic asthma	36 months	21.8%	Casale *et al.* [13]
Dupilumab	CRSwNP, eosinophilic	12 months	54%	Bagnasco *et al.* [11^▪^]
Tezepelumab	T2-high, mixed	12 months	55%	Gates *et al.* Wechsler *et al.* [15,34]
Mepolizumab	Eosinophilic asthma	12–24–36 months	52–41–73%	Bagnasco *et al.* [10^▪^]

### CHALLENGES, UNMET NEEDS, AND FUTURE DIRECTIONS

Despite significant progress, unmet needs persist. Key challenges include

(1) T2-low asthma: Limited options and poor response to current biologics.
(2) Remission biomarkers: Need for predictive and maintenance biomarkers.
(3) Biologic tapering: Criteria for safely discontinuing treatment are unclear.
(4) Cost-effectiveness: Long-term use of biologics demands economic evaluation.

An aspect to be developed in the future is the use of biomarkers to identify patients who are born with a severe form of asthma or who would benefit from early treatment with biological drugs to achieve remission. Progress should also be made in T2low asthma, which is less responsive to ICS and, at present, lacks validated biomarkers, for which we have a limited range of arms. Research into multiomic profiling, artificial intelligence-based prediction models, and new targets (e.g., IL-33, ST2, microbiome) offers promise. Trials are also underway to evaluate fixed treatment durations for biologics and re-initiation strategies upon relapse.

## CONCLUSION

The notion of asthma remission has transitioned from theoretical ideal to clinical reality, driven by mechanistic insights and targeted therapies. With personalized approaches – including biologics, early intervention, comorbidity management, and lifestyle optimization – remission is now a realistic goal for many patients.

Future strategies should emphasize early identification, treatable traits, and longitudinal monitoring. As we refine definitions and develop biomarkers, we edge closer to transforming asthma from a chronic, relapsing illness into a disease with a modifiable and potentially reversible course.

## Acknowledgements

*None.*

### Financial support and sponsorship

*None.*

### Conflicts of interest

*There are no conflicts of interest.*

## REFERENCES AND RECOMMENDED READING

Papers of particular interest, published within the annual period of review, have been highlighted as:

▪ of special interest
▪▪ of outstanding interest
